# Rational Prescribing in Primary Care (RaPP): Economic Evaluation of an Intervention to Improve Professional Practice

**DOI:** 10.1371/journal.pmed.0030216

**Published:** 2006-06-06

**Authors:** Atle Fretheim, Morten Aaserud, Andrew D Oxman

**Affiliations:** **1**Norwegian Knowledge Centre for Health Services, Oslo, Norway; George Institute for International HealthUnited States of America

## Abstract

**Background:**

Interventions designed to narrow the gap between research findings and clinical practice may be effective, but also costly. Economic evaluations are necessary to judge whether such interventions are worth the effort. We have evaluated the economic effects of a tailored intervention to support the implementation of guidelines for the use of antihypertensive and cholesterol-lowering drugs. The tailored intervention was evaluated in a randomized trial, and was shown to significantly increase the use of thiazides for patients started on antihypertensive medication, but had little or no impact on other outcomes. The increased use of thiazides was not expected to have an impact on health outcomes.

**Methods and Findings:**

We performed cost-minimization and cost-effectiveness analyses on data from a randomized trial involving 146 general practices from two geographical areas in Norway. Each practice was randomized to either the tailored intervention (70 practices; 257 physicians) or control group (69 practices; 244 physicians). Only patients that were being started on antihypertensive medication were included in the analyses. A multifaceted intervention was tailored to address identified barriers to change. Key components were an educational outreach visit with audit and feedback, and computerized reminders. Pharmacists conducted the visits. A cost-minimization framework was adopted, where the costs of intervention were set against the reduced treatment costs (principally due to increased use of thiazides rather than more expensive medication). The cost-effectiveness of the intervention was estimated as the cost per additional patient being started on thiazides. The net annual cost (cost minimization) in our study population was US$53,395, corresponding to US$763 per practice. The cost per additional patient started on thiazides (cost-effectiveness) was US$454. The net annual savings in a national program was modeled to be US$761,998, or US$540 per practice after 2 y. In this scenario the savings exceeded the costs in all but two of the sensitivity analyses we conducted, and the cost-effectiveness was estimated to be US$183.

**Conclusions:**

We found a significant shift in prescribing of antihypertensive drugs towards the use of thiazides in our trial. A major reason to promote the use of thiazides is their lower price compared to other drugs. The cost of the intervention was more than twice the savings within the time frame of our study. However, we predict modest savings over a 2-y period.

## Editors' Summary

****Background**:**

The importance of bridging the gap between research and practice, and the need to improve the prescribing practices of family doctors (general practitioners), is discussed in the Editors' Summary for an article related to this one (DOI: 10.1371/journal.pmed.0030134). However, measures to improve prescribing practice can be expensive. Economic evaluations are necessary to judge whether such measures are worth the effort.

Doctors in a Norwegian study, described in the related article, were encouraged to make more use of drugs belonging to the thiazide “family” to treat high blood pressure (hypertension). Thiazides are cheaper than other antihypertensive drugs and in the average patient at least as effective. Increasing their use should therefore save health services money, but not reduce the effectiveness of the treatment of hypertension. The study found that measures to actively encourage doctors to follow prescribing guidelines did increase the use of thiazides, but only by a small amount.

**Why Was This Study Done?:**

After having found that active promotion of guidelines can make a difference, the researchers wanted to know whether the cost of the efforts they made to encourage doctors to follow the guidelines were justified by the savings made by increased use of the cheaper drugs.

**What Did the Researchers Do and Find?:**

They calculated the money saved where the prescribing guidelines were actively promoted, and then worked out what this would amount to if the same were done in all the family practices in Norway. They found that the cost of promoting the guidelines was greater than the savings achieved during the course of their study, which lasted one year. However, their calculations show that after two years the money saved would have exceeded the costs. After that, the savings would increase every year.

**What Do These Findings Mean?:**

As far as this particular example of prescribing practice is concerned, although active promotion of guidelines increased costs in the short term, it will soon produce savings. This will not always be the case; efforts to change prescribing practice may sometimes involve the use of more effective but costlier drugs. Improving the care of patients must always be the main aim, but encouraging doctors to follow recommended guidelines on the prescribing of drugs can sometimes reduce costs, too. These issues are discussed further in a Perspective about this study (DOI: 10.1371/journal.pmed.0030229).

## Abstract 3

A significant increase in prescribing of thiazides, in preference to more expensive antihypertensives, resulted from active promotion of guidelines. The cost of the intervention was greater than the saving during the study period. Modest savings are predicted after two years.

## Introduction

Implementing high-quality clinical practice guidelines may be a way of improving clinical practice. However, the effect of implementation strategies is usually modest, and more effective interventions, such as educational outreach visits, tend to be more costly [ 1]. There are numerous trials of quality improvement strategies, but comprehensive cost–benefit or cost-effectiveness analyses are scarce. The authors of a recent systematic review of guideline implementation strategies found that “relatively few studies considered any costs other than those of treatment and its consequences” [ 2].

**Figure 1:**
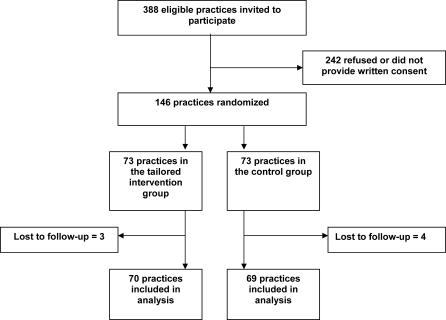
Design of Cluster-Randomized Trial

We have conducted a rigorous evaluation of a tailored intervention designed to improve prescribing of antihypertensive and cholesterol-lowering drugs in primary practice ( Figure 1) [ 3]. The intervention was multifaceted, and included (1) an educational outreach visit to clinics, during which guidelines were presented and discussed, (2) an audit and feedback on current adherence to guidelines and recommendations, and (3) a system providing computerized reminders to the physicians during patient consultations. The effectiveness of this multifaceted intervention was evaluated in a randomized controlled trial, in which the control intervention was passive dissemination of guidelines through a national medical journal [ 3]. The main outcomes analyzed in the trial were the following: (1) the proportion of prescriptions of thiazide-type diuretics to patients being prescribed antihypertensive drugs for the first time, for primary prevention of cardiovascular disease, (2) the proportion of patients for whom the level of cardiovascular risk had been estimated among all those started on antihypertensive or cholesterol-lowering treatment for primary prevention, and (3) the proportion of patients with a recorded level of cholesterol (total or low-density lipoprotein) or blood pressure satisfying the specified treatment goals among all patients on the corresponding treatment for at least 3 mo. For cholesterol we decided to also include patients on secondary prevention therapy since the treatment goals are similar.

The intervention resulted in increased prescribing of thiazides, but no significant changes in the two other main outcomes. With regards to health outcomes, the choice of antihypertensive drug has limited impact [ 4, 5], and was disregarded in our analyses. Consequently, the primary question was whether the savings on drug costs were greater than the costs of the intervention, given that the cost of thiazides is much lower than that of other antihypertensive drugs. Thus, we conducted a cost-minimization analysis comparing costs and effects of two strategies: (1) our multifaceted intervention and (2) usual care. We were also interested in knowing the costs incurred relative to the achieved changes in clinical practice, a form of cost-effectiveness analysis.

## Methods

Data collection for the economic analysis was planned before the trial was conducted. The study protocol is available via the Norwegian Knowledge Centre for Health Services Web site [ 6] and as Protocol S1.

We conducted our analyses from the perspective of the health system, i.e., those who pay for health care, since implementing these clinical practice guidelines mainly had economic implications for the funders of health care. In Norway most health care is paid for by the government and, to a minor extent, through user fees.

### Effects/Benefits

#### Dissemination and implementation of guideline.

There may be benefits resulting directly from the implementation activities, such as increased clinical knowledge or job satisfaction among the physicians, but we have no empirical data or other sound basis for estimating these effects and therefore did not include them in the analysis.

#### Changes in clinical practice as a result of guideline implementation.

The trial results demonstrated a statistically significant effect only on prescribing ( Table 1). For other outcomes we assume an absence of effect since the point estimates for the relative risks were close to one ( Table 1).

**Table 1:**
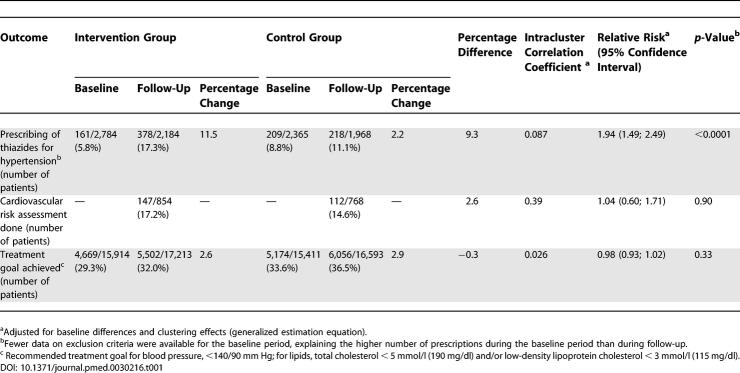
Main Results from Intervention Trial

### Costs

We included all identifiable costs related to the implementation of our intervention. The cost of guideline development was identical for both arms of the trial. Our analysis therefore focused on the incremental costs and savings of implementation beyond guideline development.

There were both nonrecurring and recurring costs related to dissemination and implementation of the guideline. Nonrecurring costs included development of software and training of outreach visitors. Recurring costs included printed materials, travels to outreach visits, salaries for the pharmacists conducting outreach visits, salary for the person making appointments for outreach visits, costs for other administrative tasks, e.g., follow-up of practices and coordination of outreach visits, and the opportunity cost of physicians' time during outreach visits. The primary cost consequence of changes in clinical practice resulting from guideline implementation was the recurring costs of drug expenditures. Data on costs and effects were collected from the 73 practices in the experimental arm of the trial, with the exception of three practices for which data collection was not possible. Outcome data, including drug costs, were also collected from the control practices.

The data sources we used for estimating resource usage are shown in Table 2.

**Table 2:**
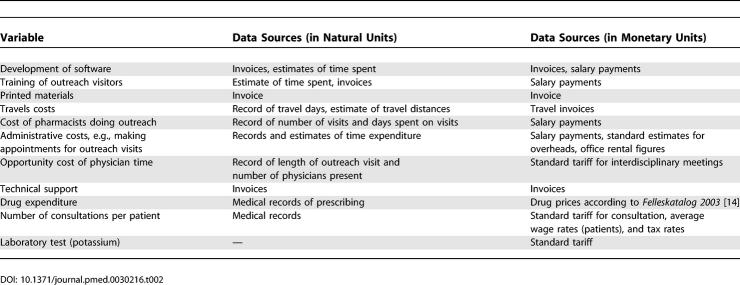
Input Variables in Economic Analysis

For each clinic, outcome data were collected for 1 y after the outreach visit took place. The primary analysis is based on these data and assumes that all costs and all benefits occur within the same year; thus, we can disregard discounting. However, discounting was included in the analyses in which effects were modeled beyond 1 y. We used a discounting rate of 4%, in accordance with guidelines from the Norwegian Ministry of Finance. Value added tax was excluded from the cost figures in the analyses. Salary costs included payroll taxes, social costs, and a 40% institutional overhead.

All figures are expressed in 2002 United States dollars, using the 2002 average exchange rate from Norwegian kroner [ 7].

### Analysis

#### Cost minimization.

For the cost-minimization analysis we included all costs related to the intervention, including drug costs ( Protocol S2, Equation 1). For the usual-care group we assumed no intervention costs.

We estimated drug costs by multiplying the total number of prescriptions of antihypertensives to patients started on treatment in the intervention group during the study period by the difference in cost per prescription between the two groups ( Protocol S2, Equation 2). We adjusted for baseline differences ( Protocol S2, Equation 3).

#### Cost-effectiveness.

We defined cost-effectiveness as the cost incurred per additional patient started on a thiazide rather than another antihypertensive drug; i.e., an incremental cost-effectiveness ratio of intervention versus usual care ( Protocol S3, Equation 1).

We did not include savings resulting from changes in prescribing patterns in the cost-effectiveness analysis since our objective was to produce an estimate with relevance across interventions that target prescribing habits, independent of whether a change leads to higher or lower drug expenditures.

The number of patients started on thiazides because of the intervention was calculated by multiplying the absolute between-group difference in proportion of patients started on thiazides by the total number of patients started on antihypertensive treatment in the intervention group ( Protocol S3, Equation 2). We adjusted for baseline differences between the groups ( Protocol S3, Equation 3).

### Assumptions

We assumed there were no effects on health outcomes or on the use of health services for two reasons. Firstly, the association between choice of antihypertensive drug and health outcomes is marginal [ 4, 5]. Secondly, the average number of consultations per patient started on antihypertensive therapy was the same (4.0) in both groups in the study.

We did not have access to the number of tablets per prescription. Based on data we recently collected in a similar population of practices and patients [ 8], we assumed that one prescription represented a standard package of 100 tablets (or the available package size closest to this), which was dispensed twice. We assumed that the strength of the prescribed tablets corresponded to one defined daily dosage [ 9].

We excluded costs related to patient time since we found it unlikely that the intervention had an effect on this.

### Sensitivity Analysis

We performed univariate sensitivity analyses with adjusted values for all variables we believed could impact on our findings.

### Scaling Up to Nationwide Implementation

We used our findings in a model to estimate the economic consequences of scaling up the intervention to a national outreach program. The model was based on the following prespecified assumptions: (1) 90% of all practices in Norway covered (using a total number of practices of 1,567 [A. Taraldset, Norwegian Medical Association, personal communication]), (2) same effect on prescribing as observed in the trial, (3) same pattern of prescribing as in the study population, and (4) higher travel costs than in the trial due to longer distances to cover.

In order to produce a best estimate for the cost of a national program, we added the following assumptions: software development costs same as in trial, salary for pharmacists set to average salary wage among pharmacy pharmacists in Norway, i.e., about 50% higher than pharmacist salaries in the trial [ 10], each outreach visitor covers 200 practices per year, opportunity costs of physicians included, average price of thiazides and non-thiazides unchanged from trial, effect on drug costs sustained for 2 y (i.e., the patients started on antihypertensives during the first year continue taking the same drugs during the full second year, and prescribing of antihypertensives for new patients in year two follows the same pattern as in year one), one potassium test ordered per additional patient started on thiazides (US$3.60 per test), and travel costs doubled compared to trial.

Finally, we also tested the robustness of our national program model by adjusting the values of most variables in univariate sensitivity analyses.

## Results

The estimated costs for the various classes of antihypertensive drugs are shown in Table 3, including the total number of prescriptions (5,191 in intervention group) and costs per prescription (net change: US$7.47). Thus, savings on drugs were estimated to be US$38,773 for the 70 practices in the intervention group over 1 y.

**Table 3:**
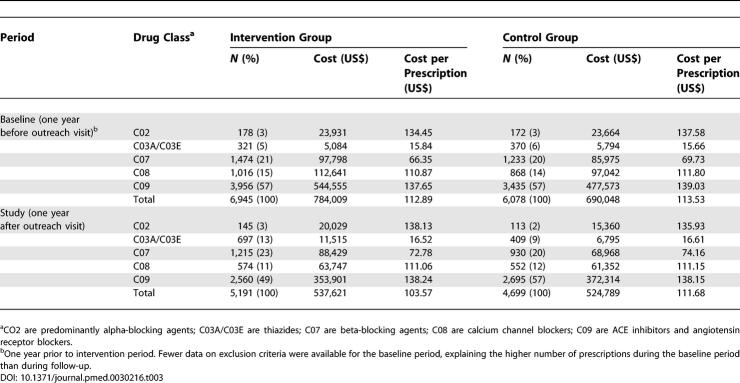
Number of Prescriptions and Drug Costs during Study and Baseline Periods

All costs and savings related to the intervention are presented in Table 4. The net cost (cost minimization) of implementing the intervention in our study population was US$53,395, corresponding to US$763 per practice.

**Table 4:**
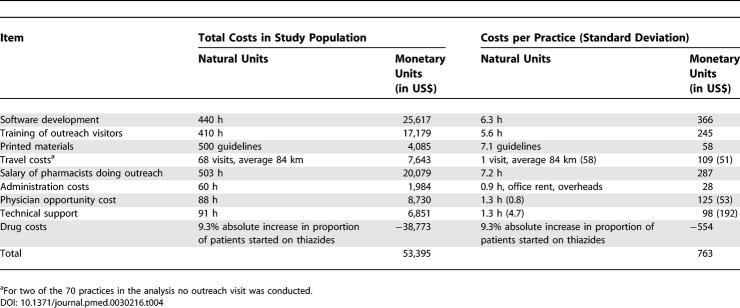
Costs Related to the Intervention

The trial results demonstrated an effect of the intervention on the prescribing of thiazides, with an absolute increase of 9.3% ( Table 1, adjusted for baseline differences). The total number of patients started on medication in the intervention group was 2,184; 203 patients (9.3% of 2,184) were started on thiazides rather than another drug because of the intervention. The cost of the intervention, excluding savings on drugs, added up to US$92,168. Consequently, the cost-effectiveness of the intervention, i.e., the cost per additional patient started on thiazides, was US$454.

### Sensitivity Analyses

Adjusting the assumptions in our main analysis had an impact on the estimated cost of the intervention, but the costs remained higher than the savings in all the univariate sensitivity analyses we conducted, except when we assumed that the effect of the intervention would be sustained for a second year ( Table 5).

**Table 5:**
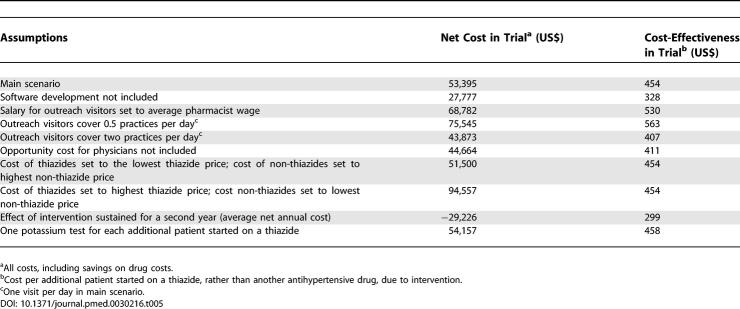
Univariate Sensitivity Analyses

### Model for Nationwide Implementation

In our model for estimating the economic impact of a national outreach program, the savings exceeded the costs, largely because of our assumption of the effect being sustained over 2 y ( Table 6). The total annual savings amounted to US$761,998, or US$540 per practice. The savings also exceeded the costs in all but two of the univariate sensitivity analyses we conducted on this model ( Table 7). The cost-effectiveness was estimated to be US$183 per additional patient started on a thiazide.

**Table 6:**
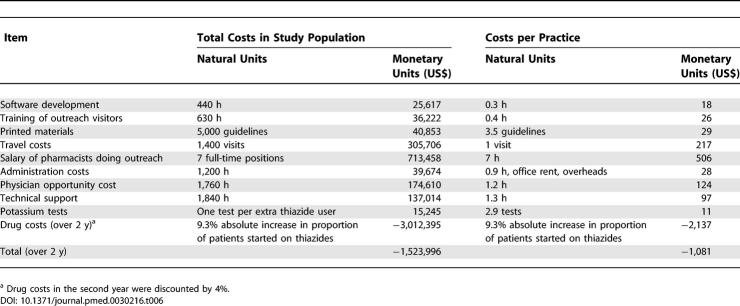
Costs Related to the Intervention in Model for Scaling Up to National Program

**Table 7:**
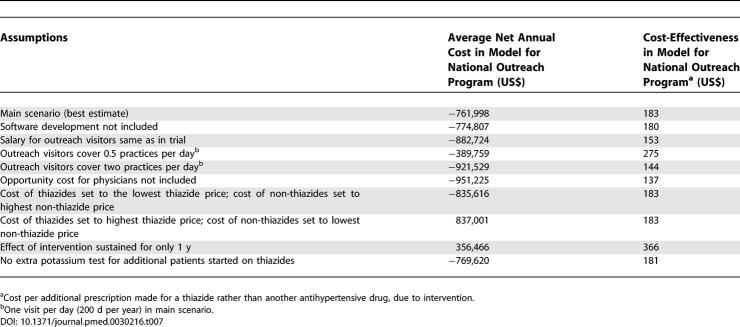
Univariate Sensitivity Analyses in Model for Scaling Up to National Program

## Discussion

We found a considerable shift in the prescribing of antihypertensive drugs towards the use of thiazides in our trial. In our economic analysis of the trial data we found that reduced drug expenditures due to increased use of thiazides did not outweigh the costs of the intervention. However, assuming that the effect is sustained for a second year, the picture changes and the intervention can be expected to lead to savings. We predict modest savings within 2 y if the intervention is implemented in a national program.

The key issue in determining the cost-saving potential of the intervention is whether the intervention effect will be sustained beyond a year, which was the duration of the trial. There are two good reasons to believe that this will be the case. Firstly, it is reasonable to believe that most patients who start using thiazides will continue to use these drugs rather than the more expensive alternatives, although many patients will be started on additional drugs regardless of the first drug given. This may translate into savings in subsequent years. Secondly, it is likely that the intervention will continue to influence physicians' choice of antihypertensive medication for new patients beyond the first year after the outreach visit took place. In the trial, the rate of thiazide prescribing to new patients was stable throughout the study period in the intervention group, with the effect showing no sign of waning over time.

Whether it is reasonable to assume that the intervention effect will be sustained beyond 2 y is less certain. Firstly, the pharmaceutical market is dynamic, with prices constantly changing and the regular introduction of new products. Secondly, countermeasures from pharmaceutical companies may be anticipated if health authorities launch national outreach programs aimed to influence physicians' prescribing.

There are few other economic analyses with which we can compare our results. Whether a cost of US$454 for changing a prescribing decision is a good or bad result is unclear. The intervention also targeted other behaviors, such as physicians' use of risk assessment tools, and had little or no impact on these. Thus, for these behaviors the intervention would be more costly but no more effective, i.e., it would represent an inefficient use of resources.

In light of these results, policy-makers need to consider alternative strategies if they wish to influence prescribing more cost-effectively. Recently, authorities in Norway made thiazides mandatory as first-choice medication if patients were to have drug costs reimbursed through the national health insurance system. The effectiveness of this strategy is under evaluation. Another alternative is establishing a maximum level of reimbursement for a group of drugs assumed to be therapeutically equivalent (reference pricing). This strategy has been thoroughly evaluated in British Columbia, Canada, for one class of antihypertensive drugs (angiotensin-converting enzyme inhibitors), and was found to lead to substantial savings on health budgets [ 11]. However, applying one reference price for all antihypertensive drugs may be more difficult to implement and has not, to our knowledge, been evaluated rigorously.

There is some debate about whether the choice of antihypertensive drug has an impact on health outcomes, which again may have economic implications. For some drug classes, e.g., alpha-blocking agents [ 12], there is sound evidence of inferiority compared to other classes, while for other drug classes, e.g., angiotensin receptor blockers, it is unclear whether the therapeutic effects are as good as for other classes [ 13]. It could be argued that an increased use of thiazides would lead to improved health outcomes [ 5], but the superiority of thiazides over other drugs is small in any case, and is not likely to have had much impact on the present analysis.

There are weaknesses with our study, mainly related to the many assumptions we had to make. However, almost all the sensitivity analyses we conducted, including our suggested best estimate, point in the same direction: if the intervention effect is sustained for a second year, modest savings can be expected.

We have assumed that the intervention had no impact on the volume of prescribing of antihypertensive medication. The trial data on prescribing volumes may be interpreted differently since the absolute difference between prescribing volumes in the experimental and control groups differed between the baseline period (one year before the outreach visit) and the study period (one year after the outreach visit) ( Table 3). However, the ratios of prescribing volumes during the study period versus during the baseline period were similar for the two groups, and the observed difference may well be a chance finding. It is also highly unlikely that taking this finding into account would change the conclusions.

This study is one of very few comprehensive economic analyses of quality improvement strategies that include educational outreach visits. The lack of economic evaluations has been pointed out by others, and our findings illustrate that such analyses are important for well-informed decision-making.

### Conclusions

The findings from our trial were similar to previous studies of outreach visits aimed at influencing prescribing patterns. Despite savings due to increased use of less expensive drugs, the intervention resulted in a net cost within the 1 y that the trial lasted. However, it is likely that the intervention would lead to modest savings over a 2-y period.

Publicly funded educational outreach programs should perhaps be targeted primarily at improving quality of care rather than costs of health care, given the cost of outreach visits, competition from drug companies, and the availability of alternative strategies for controlling costs (such as reference pricing, pre-authorization, and restrictions on reimbursement).

## Supporting Information

Protocol S1 Study Protocol (112 KB PDF)

Protocol S2 Cost-Minimization Equations (82 KB DOC)

Protocol S3 Cost-Effectiveness Equations (24 KB DOC)
