# Supplementary material for: Rational Prescribing in Primary Care (RaPP): Economic Evaluation of an Intervention to Improve Professional Practice
Source: PLoS Med. 2006 Jun 6;3(6):e216. doi: 10.1371/journal.pmed.0030216 (PMC1472698; doi:10.1371/journal.pmed.0030216)
Supplement: Supplementary file 1 [file pmed.0030216.sd001.pdf]

Nasjonalt kunnskapssenter for helsetjenesten

Norwegian Knowledge Centre for Health Services

Economic analyses of an intervention to improve prescribing of antihypertensive and cholesterol-lowering drugs

Notat: Forskningsprotokoll, 3 mai 2005

ISBN 82-8121-047-8

Tittel	Economic analyses of an intervention to improve prescribing of antihypertensive and cholesterol-lowering drugs
Institusjon	Nasjonalt kunnskapssenter for helsetjenesten
Prosjektansvarlig	Atle Fretheim
Forfatter	Atle Fretheim, Morten Aaserud, Andrew D. Oxman
ISBN	82-8121-047-8
Forsknings-protokoll	3. mai 2005
Prosjektnummer	114
Antall sider	11

Nasjonalt kunnskapssenter for helsetjenesten fremskaffer og formidler kunnskap om effekt, nytte og kvalitet av metoder, virkemidler og tiltak innen alle deler av helsetjenesten.

Kunnskapssenteret er formelt et forvaltningsorgan under Sosial- og helsedirektoratet. Det har ingen myndighetsfunksjoner og kan ikke instrueres i faglige spørsmål.

Nasjonalt kunnskapssenter for helsetjenesten

Oslo, mai 2005

Background

Implementing high-quality clinical practice guidelines may be a way of improving clinical practice. However, the effect of implementation strategies is usually modest, and more effective interventions, such as educational outreach visits, tend to be the costliest.[1] Despite there having been conducted numerous trials of quality improvement strategies, comprehensive cost-benefit or cost-effectiveness analyses are scarce. The authors of a recent systematic review of guideline implementation strategies found that “relatively few studies considered any costs other than those of treatment and its consequences”.[2]

We have conducted a rigorous evaluation of a tailored intervention designed to improve prescribing of antihypertensive and cholesterol-lowering drugs in primary practice (see fig 1).[3] The intervention was multifaceted, and included an educational outreach visit to clinics where guidelines were presented and discussed; an audit and feedback of current adherence to guidelines-recommendations; and a system providing computerised reminders to the physicians during patient consultations. The effectiveness of the multifaceted intervention was evaluated through a randomised controlled trial design, where the control intervention was passive dissemination of guidelines through a national medical journal.[3] The main outcomes in the trial were:

The proportion of prescriptions of other antihypertensives than thiazides to patients being prescribed antihypertensive drugs for the first time.

The proportion of patients where the level of cardiovascular risk had not been estimated among all those started on antihypertensive or cholesterol lowering treatment

Proportion of patients with a recorded level of cholesterol (total or LDL) or blood pressure not satisfying the specified treatment goals, among all patients on the corresponding treatment for at least three months. For cholesterol we decided to also include patients on secondary prevention therapy since the treatment goals are similar

The intervention led to increased prescribing of thiazides but no significant changes in the two other main outcomes. We expect no health consequences of the changes in prescribing patterns, thus the primary question is whether the savings on drug-costs are greater that the costs of the intervention, (cost- minimisation analysis). We are also interested in knowing the costs incurred relative to the achieved changes in clinical practice, a form of cost-effectiveness analysis.

Methods

We will conduct our analyses from the perspective of the health system, i.e. those who pay for health care, since implementing these clinical practice guidelines mainly has economic implications for the funders of health care. In Norway most health care is paid through taxation and to a minor extent through user-fees.

There are several stages in the introduction and use of guidelines that may be included in an economic evaluation[4]:

- Development of guidelines
- Dissemination and implementation of the guidelines
- Changes in clinical practice as a result of guideline implementation

In our analysis we will ignore costs and effects related to guideline development. There are several reasons for this. Firstly, our guidelines were developed specifically for this project. They do not represent a useful estimate of the expected costs of guideline-development within an established program. Secondly, guidelines are widely developed independently by a variety of organisations. Finally, the cost of the guideline development is identical for both arms of the trial. Our analysis therefore focuses on the marginal costs and savings of implementation beyond guideline development.

Effects/benefits

Dissemination and implementation of guideline

There may be benefits resulting directly from the implementation activities, such as increased clinical knowledge or job satisfaction among the physicians, but we have no empirical data or other sound basis for estimating these effects and will therefore not include them in the analysis.

Changes in clinical practice as a result of guideline implementation

These benefits correspond to the findings from the trial, but will be limited to the pre-specified primary outcomes. The results from the trial demonstrated a statistically significant effect on prescribing, but not for the other primary outcomes. The relative risk for these outcomes where very close to 1, and the 95% confidence intervals around the effect-estimates were relatively narrow. Consequently, in the analysis we choose to assume an absence of effect on main outcomes other than those

related to prescribing. We will include secondary outcomes in the sensitivity analysis, if there are wide confidence intervals around the effect estimates.

Costs

All costs will be reported in monetary as well as natural units. An overview of all included costs and data sources are found in Table 1. The following will be included:

Costs related to dissemination and implementation of guideline

Non-recurring costs:

- Development of software
- Training of outreach-visitors

Recurring costs:

- Printed materials
- Travels to outreach visits
- Cost for pharmacist conducting outreach visits
- Cost for person making appointments for outreach visits
- Costs for other administrative tasks, e.g. follow-up of practices and co-ordination of outreach visits
- Opportunity cost of physicians' time during outreach visit

Cost-consequences of changes in clinical practice resulting from guideline implementation

Recurring costs:

- Drug expenditures
- Number of consultations

For each clinic, outcome data was collected for one year after the outreach visit had taken place. The primary analysis is based on these data and assumes that all costs and all benefits occur within the same year, thus we can disregard discounting.
We have excluded costs related to patient time since we find it unlikely that the intervention had an effect on this.

Analysis

For the cost-effectiveness analysis we will calculate the cost incurred per patient started on a thiazide rather than another antihypertensive drug. We will not include drug-expenditures in this analysis. This would have entailed double counting because the changes in drug costs are a direct consequence of changes in prescribing. To adjust for baseline-differences we will calculate the net change in prescribing of thiazides, which we will use as effect-estimate:

Effect-estimate = Net change

= (Post-intervention – Pre-intervention) $_{\text{intervention-group}}$ - (Post-intervention – Pre-intervention) $_{\text{control group}}$

The effect-size will be applied on the total number of patients started on antihypertensive treatment in the experimental group, thus providing us with an estimated number of patients that are started on a thiazide rather than a non-thiazide due to the intervention:

Cost-effectiveness = $\dfrac{\sum \text{costs}_{\text{intervention}} - \sum \text{costs}_{\text{control}}}{(\mathit{Effect\text{-}estimate}) \times N_{\text{Prescriptions}}}$

For the cost-minimisation analysis we will include all costs and savings:

Cost-minimisation
$= (\sum \text{costs}_{\text{intervention}} - \sum \text{costs}_{\text{control}}) - (\sum \text{savings}_{\text{intervention}} - \sum \text{savings}_{\text{control}})$

We will adjust for baseline differences between the intervention and control groups when estimating costs and savings.

We will perform sensitivity analyses with adjusted values for several variables – see Table 1 for details. This will be done for both the cost-effectiveness and the cost-minimisation analyses.

One of the variables involves the time horizon for the effect of the intervention. In the main analysis we only include the measured effects during the first year after the intervention. In the sensitivity analysis we will expand this to also include effects during the second year. We will calculate the effect-size as a function of time during the first year after the outreach visit. If the regression coefficient is positive we will use the point estimate after one year as the estimated effect for the following year. If the regression coefficient is negative, we will assume the downward slope continues through the second year, until the costs of prescribing reaches

the same level as in the control group. We will use a discount rate of 4 % for the second year, in accordance with guidelines from the Norwegian Ministry of Finance.[5]

The rationale for excluding development costs for software in the sensitivity analysis is that the software will be available for use in future programs and/or that we may assume that the outreach visit was the essential element of the multifaceted intervention – not the software package.

Assumptions

We assume that health outcomes are not affected by the choice of antihypertensive[6;7].

We do not have access to the number of tablets per perscription. We will estimate this figure based on:

- The number of days between consecutive prescriptions for each specific drug in the trial
- National sales figures for antihypertensive drugs broken down to class and package-size
- The fact that most drugs are sold in packages of 28 or 100 tablets

Scaling up to nationwide implementation

We will estimate the cost-effectiveness of the intervention modelled to implementation at national level, using these assumptions:

- 90% of all practices in Norway covered
- Same effect-size as found in trial
- Same pattern of prescribing as in study population
- Travel costs higher than in trial (We will collect information on the cost of outreach visits conducted nationally by pharmaceutical representatives. The findings will be related to the costs of outreach-activities from the trial, and based on this we will produce a best-estimate for travel costs.)

The estimated number of practices covered by each outreach visitor per year will be estimated based on experiences from the conduct of the trial and on information gathered from pharmaceutical companies that run national outreach programs

Discussion

The only implications of changes in prescribing that we have included in our analysis are on drug costs and the frequency of consultations. There is some debate around whether the choice of antihypertensive drug has an impact on health outcomes, which again may have economic implications. For some drug-classes there is sound evidence of inferiority compared to other classes, e.g. alpha-blocking agents[8], and for some drug-classes, e.g. angiotensin receptor blockers, it is unclear whether the therapeutic effects are as good as for other classes.[9] It could be argued that an increased use of thiazides would lead to improved health outcomes[7], but the superiority of thiazides over other drugs is small and will not be included in this analysis.

Competing interests

AF and ADO were responsible for the RaPP-trial, which was an evaluation of the intervention in question. MA has previously carried out short term pharmacoeconomic projects for the National Insurance Service and the Norwegian Medicines Agency. From 1997 to 1999 he worked for a private company, Brevreklame, doing market research for pharmaceutical firms in Norway.

Authors' contributions

AF made the first draft, which was developed further through discussion and input from MA and ADO.

Reference List

1. Grimshaw JM, Shirran L, Thomas R, Mowatt G, Fraser C, Bero L *et al*. Changing provider behavior: an overview of systematic reviews of interventions. *Med Care* 2001;**39**:II2-45.

2. Grimshaw JM, Thomas RE, MacLennan G, Fraser C, Ramsay CR, Vale L *et al*. Effectiveness and efficiency of guideline dissemination and implementation strategies. *Health Technol.Assess.* 2004;**8**:iii-72.

3. Fretheim A, Oxman A, Treweek S, Bjørndal A. Rational Prescribing in Primary Care (RaPP-trial). A randomised trial of a tailored intervention to improve prescribing of antihypertensive and cholesterol-lowering drugs in general practice [ISRCTN48751230]. *BMC Health Services Research* 2003;**3**:5.

4. McIntosh E. Economic evaluation of guideline implementation strategies. pp 77-98. 1999.

5. Behandling av diskonteringsrente, risiko, kalkulasjonspriser og skattekostnad i samfunnsøkonomiske analyser. 22-12-1999. Norwegian Ministry of Finance.

6. Blood Pressure Lowering Treatment Trialists' Collaboration. Effects of different blood-pressure-lowering regimens on major cardiovascular events: results of prospectively-designed overviews of randomised trials. *Lancet* 2003;**362**:1527-35.

7. Psaty BM, Lumley T, Furberg CD, Schellenbaum G, Pahor M, Alderman MH *et al*. Health outcomes associated with various antihypertensive therapies used as first-line agents: a network meta-analysis. *JAMA* 2003;**289**:2534-44.

8. Major cardiovascular events in hypertensive patients randomized to doxazosin vs chlorthalidone: the antihypertensive and lipid-lowering treatment to prevent heart attack trial (ALLHAT). *JAMA* 2000;**283**:1967-75.

9. Verma S,.Strauss M. Angiotensin receptor blockers and myocardial infarction. *BMJ* 2004;**329**:1248-9.

Figure 1. Design of cluster-randomised controlled trial

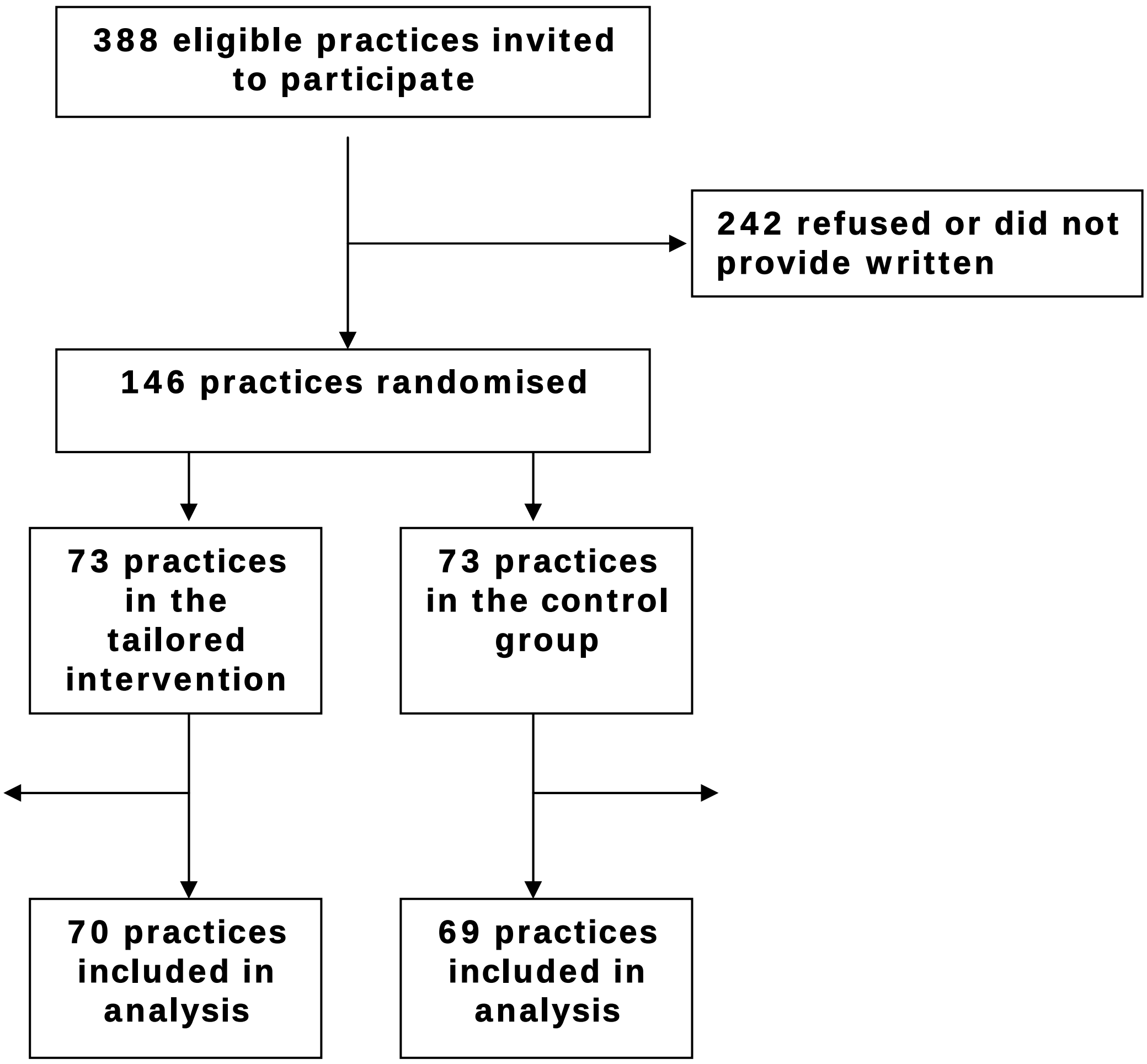

Table 1. Input variables in economic analysis

Variable	Data-source (natural units)	Source for monetary units	Sensitivity analysis
Development of software	Invoices, estimates of time spent	Invoices, salary payments	Exclusion of development cost
Training of outreach visitors	Estimate of time spent	Salary payments	Mean or median wage for pharmacists in Norway
Printed materials	Invoice	Invoice	-
Travels costs	Estimate of distance to practices; record of number of travel days	Travel invoices	-
Cost of pharmacists doing outreach	Record of number of visits and days spent on visits	Salary payments	Mean or median wage for pharmacists in Norway; 0.5 to 2 visits per day
Cost of making appointments	Record of time expenditure	Salary payments	95 % CI of time expenditure
Cost of other administrative tasks	Estimated time expenditure, cost of office for one person	Salary payments; standard estimates for overheads	Mean or median wage for executive officers in the public sector in Norway
Opportunity cost of physician time	Record of length of outreach visit and number of physicians present	Standard tariff for interdisciplinary meetings	95 % CI of physician time; Cost adjusted to zero (assuming visit during lunch, i.e. with no real loss of income for physicians)
Technical support	Invoices	Invoices	95 % CI of time spent per practice; Mean or median wage for IT-consultants in Norway
Drug expenditure	Medical records of prescribing	"Felleskatalogen 2003" (list of drugs and prices)	95 % CI of intervention-effect; Time extended (proportion of effect maintained in following year); Adjustments for variations in prices (only most expensive thiazides/cheapest non-thiazides; only cheapest thizide/most costly non-thiazide)
Number of consultations Per patient	Medical records	Standard tariff for consultation Average wage-rates (patients) and tax-rates	95 % CI of number of consultations (only if data show statistically significant difference)
Laboratory test (potassium)	-	Standard tariff	One test per extra patient started on thiazide
