# Supplementary material for: Rational Prescribing in Primary Care (RaPP): Economic Evaluation of an Intervention to Improve Professional Practice
Source: PLoS Med. 2006 Jun 6;3(6):e216. doi: 10.1371/journal.pmed.0030216 (PMC1472698; doi:10.1371/journal.pmed.0030216)
Supplement: Supplementary file 2 [file pmed.0030216.sd002.doc]

## Protocol 1. Cost-minimization equations

**Equation 1:**

Costs _intervention group_ - Costs _control group_

= (Σ Intervention costs _intervention group_ - Σ Intervention costs _control group_)

+ (Σ Drug costs _intervention group_ - Σ Drug costs _control group_)

Intervention costs in the control group are zero.

**Equation 2:**

(Σ Drug costs _intervention group_ - Σ Drug costs _control group_)

*= (Number of prescriptions _intervention group_ × Cost per prescription _intervention group_) – (Number of prescriptions _control group_ × Cost per prescription _control group_)*

We assume

*Number of prescriptions _intervention group_ = Number of prescriptions _control group_*

(This assumption is addressed in the Discussion-section)

Thus,

(Σ Drug costs _intervention group_ - Σ Drug costs _control group_)

*= Number of prescriptions _intervention group_ × (Cost per prescription _intervention group_ – Cost per prescription _control group_)*

**Equation 3:**

Adjusting for baseline-differences:

(Cost per prescription _intervention group_ – Cost per prescription _control group_)

= (Cost per prescription _study period_ – Cost per prescription _baseline period_) _intervention group_

- (Cost per prescription _study period_ – Cost per prescription _baseline period_) _control group_
