# Supplementary material for: Rational Prescribing in Primary Care (RaPP): Economic Evaluation of an Intervention to Improve Professional Practice
Source: PLoS Med. 2006 Jun 6;3(6):e216. doi: 10.1371/journal.pmed.0030216 (PMC1472698; doi:10.1371/journal.pmed.0030216)
Supplement: Supplementary file 3 [file pmed.0030216.sd003.doc]

## Protocol 2. Cost-effectiveness equations

**Equation 1:**

*Incremental cost-effectiveness ratio*

*= Incremental cost / Incremental effect*

*= (Σ Intervention costs _intervention group_ - Σ Intervention costs _control group_)*

*/ (Patients started on thiazides _intervention group_ - Patients started on thiazides _control group_)*

Intervention costs in the control group are zero.

**Equation 2:**

*(Patients started on thiazides _intervention group_ - Patients started on thiazides _control group_)*

*= (Number of patients started on antihypertensives _intervention group_ × Proportion started on thiazides _intervention group_) – (Number of patients started on antihypertensives _control group_ × Proportion started on thiazides _control group_)*

We assume

*Number of patients started on antihypertensives _intervention group_*

*= Number of patients started on antihypertensives _control group_*

(This assumption is addressed in the Discussion-section)

Thus,

*(Patients started on thiazides _intervention group_ - Patients started on thiazides _control group_)*

*= Number of patients started on antihypertensives _intervention group_ × (Proportion started on thiazides _intervention group_ – Proportion started on thiazides _control group_)*

**Equation 3:**

Adjusting for baseline-differences:

Proportion started on thiazides _intervention group_ – Proportion started on thiazides _control group_

= (Proportion started on thiazides _study period_ – Proportion started on thiazides _baseline period_) _intervention group_

- (Proportion started on thiazides _study period_ – Proportion started on thiazides _baseline period_) _control group_
